# Structural changes in adolescent mental health networks from the pandemic to the post-pandemic period: a network comparison study

**DOI:** 10.1186/s13034-025-01021-0

**Published:** 2026-01-31

**Authors:** Jeong Yeop Whang, Heeyeon Kim

**Affiliations:** 1 Yonsei University College of Medicine, Seoul, Republic of Korea; 2 Department of Psychiatry, Yongin Severance Hospital, Yonsei University College of Medicine, Yongin-si, Republic of Korea; 3 Institute of Behavioral Science in Medicine, Yonsei University College of Medicine, Seoul, Republic of Korea

**Keywords:** Adolescents, Mental health, Network analysis, COVID-19, Suicidality, Smartphone overdependence, Sleep

## Abstract

**Objective:**

To examine the structural changes in adolescent mental health symptom networks between the COVID-19 pandemic and post-pandemic periods in South Korea using nationally representative data and network analysis.

**Methods:**

We analyzed data from the Korean Youth Risk Behavior Survey collected during the pandemic (2020; *N* = 46,387) and post-pandemic (2023; *N* = 48,936). Thirty theoretically selected variables were included, covering anxiety symptoms, smartphone overdependence, sleep quality, suicidality, substance use, and demographic characteristics. Regularized partial correlation networks were estimated using LASSO with extended Bayesian information criterion selection. Network comparison tests were used to assess structural differences, and three centrality indices (strength, betweenness, and closeness) were calculated. Sex-stratified networks were also examined to explore potential differences in symptom configurations.

**Results:**

Symptom networks showed marked reorganization from the pandemic to the post-pandemic period. Suicidal ideation and planning demonstrated higher centrality in the post-pandemic network, particularly within the female subgroup, whereas suicide attempts showed reduced centrality. Irritability became more prominent within the anxiety domain among females, while worry-related symptoms gained centrality among males. Family-related smartphone conflict and sleep timing variables showed stronger integration within the female network, suggesting greater integration into the mental health network and heightened vulnerability among girls in the post-pandemic context.

**Conclusion:**

Adolescent mental health networks in South Korea have changed substantially between the pandemic and post-pandemic periods. Beyond changes in prevalence, network restructuring highlighted shifts in how symptoms relate to one another, with notable sex-specific patterns. These findings underscore the value of symptom-level, network-based approaches for understanding post-pandemic mental health and emphasize the need for sex-sensitive and developmentally informed screening and interventions.

**Supplementary Information:**

The online version contains supplementary material available at 10.1186/s13034-025-01021-0.

## Introduction

As of May 5, 2023, the World Health Organization has declared that COVID-19 no longer constitutes a Public Health Emergency of International Concern [1]. Adolescents undergoing critical biological, psychological, and social development were particularly vulnerable to widespread disruptions during the pandemic. Social distancing, school closures, and the sudden loss of peer interaction and daily structure curtailed access to protective resources, such as education, physical activity, and social support [2, 3], while fear of contagion and family stress intensified emotional distress [4].

Although many studies have documented elevated rates of depression, anxiety, and other mental health concerns during the pandemic, considerably fewer studies have addressed the consequences that followed the lifting of restrictions : Did adolescent mental health recover? Emerging evidence suggests a more complicated picture. Some symptoms, such as reduced physical activity, may have gradually improved, while others, including sadness, suicidal ideation, and emotional dysregulation, may have persisted during the post-pandemic era [5, 6]. Moreover, longitudinal studies have indicated that adolescents’ subjective experiences of disrupted learning and peer connections, rather than the lockdown duration itself, are key predictors of lingering emotional and behavioral difficulties [7–10]. However, few studies have systematically investigated how the configuration of mental health symptoms changed during this transition, particularly in terms of symptom interconnectivity and network structure.

Along with these psychosocial shifts, adolescents’ engagement with digital technology, especially smartphones and social media, has augmented during and after the pandemic. Before COVID-19, excessive smartphone use was associated with increased anxiety, loneliness, and emotional dysregulation [11, 12]. During and after the pandemic, high screen time has been linked to ongoing emotional difficulties, making digital behavior a potential contributor to persistent mental health risks [8, 11].

From a transdiagnostic perspective, these domains are not independent phenomena but form an interconnected system in adolescence. Sleep disturbance and emotion regulation difficulties have been identified as core processes that increase vulnerability to anxiety and depression, heighten suicidal thoughts and behaviors, and promote maladaptive coping, including substance use [13, 14]. Problematic smartphone use further interacts with these pathways: excessive or compulsive use is associated with poorer sleep quality and shorter sleep duration, which in turn amplify emotional dysregulation and internalizing symptoms [15, 16]. Substance use may offer short-term relief from negative affect but ultimately worsens sleep and cognitive control. These mutually reinforcing processes highlight the need to examine these domains together within an integrated analytic framework.

Traditional latent-variable approaches summarize shared variance across symptoms but cannot illuminate how specific symptoms activate or reinforce others—a process that may shift during major societal transitions [17]. Network analysis addresses this limitation by identifying the most central or influential symptoms within the system, thereby providing insight into how distress may propagate and which symptoms may serve as efficient intervention targets [18–21]. Such methods are particularly relevant in adolescent populations, where comorbidities are common and symptom profiles are context sensitive, heterogeneous, and highly responsive to environmental stressors, such as the COVID-19 pandemic [22].

Sex differences are highly relevant to adolescent mental health and justify separate network models. Girls consistently exhibit higher rates of internalizing symptoms and suicidal ideation/attempts [23], whereas boys more often display externalizing behaviors and substance use. Sleep problems and smartphone overdependence also show a stronger burden among girls [24]. Importantly, recent network studies indicate that these sex differences extend beyond prevalence to differences in how symptoms co-occur and reinforce one another [25, 26]. Accordingly, sex-stratified networks are essential not only to compare overall symptom levels but to determine whether boys and girls exhibit distinct configurations of symptom interrelationships in the post-pandemic context.

In this study, we aimed to examine how adolescent mental health networks changed between the COVID-19 pandemic (2020) and post-pandemic (2023) periods using nationally representative data from the Korean Youth Risk Behavior Survey. By applying network analysis, we focused on key domains including anxiety, suicidal ideation and behavior, smartphone overdependence, sleep, and substance use, which are factors known to interact in complex ways during adolescence. We also conducted sex-stratified analyses to explore sex-specific shifts in the symptom structures. By identifying changes not only at the symptom level, but also in how symptoms relate to one another, this study offers insights into the evolving architecture of adolescent psychopathology and helps identify new targets for early, personalized, and context-sensitive interventions.

## Methods

### Data source and participants

Data were drawn from the 2020 and 2023 Korean Youth Risk Behavior Web-based Survey (KYRBS), an annual, self-administered, nationally representative survey of middle and high school students conducted by the Korea Disease Control and Prevention Agency [27, 28]. The KYRBS collects information on health-related behaviors, such as diet, physical activity, substance use, mental health, Internet addiction, and psychosocial factors.

To represent the pandemic and post-pandemic periods, we used data from 2020 (*N* = 54,948) and 2023 (*N* = 52,880), which included 175 and 160 survey items, respectively. We selected 30 variables related to anxiety, smartphone use, and mental health based on their relevance to adolescent psychological functioning. These variables were chosen based on their established relevance to core domains of adolescent mental health (suicidality, affective and anxiety symptoms, sleep, smartphone overuse, and substance use). Variables with high missingness, inconsistent measurement across years, or very high intercorrelation (e.g., family background, second-hand smoking, and physical-activity items) were excluded to ensure measurement consistency and reliable network estimation. After excluding participants with missing values, the final analytical samples were 46,387 (2020) and 48,936 (2023). A detailed flowchart summarizing variable selection, exclusion criteria, and the final analytical samples is provided in Supplementary Figure S1.

### Measures

Thirty variables representing the key domains of adolescent mental health were selected for network analysis.

1. *Demographic variables* included age (continuous) and sex (binary: 0 = male; 1 = female). Sex was included in the overall mixed graphical model as a binary categorical variable to examine population-level associations with mental-health–related nodes. Although demographic variables appear visually identical to symptom nodes in an undirected network, sex was conceptualized as a non-activatable demographic attribute rather than a psychological symptom, meaning that its associations reflect fixed group differences rather than symptom-driven activation. Separate sex-stratified networks were additionally estimated to examine whether symptom-to-symptom associations differed between boys and girls.
2. *Anxiety symptoms* were assessed using the 7-item Generalized Anxiety Disorder scale (GAD-7), with each item rated from 0 (“not at all”) to 3 (“nearly every day”), reflecting symptom frequency over the past two weeks. The items were in the following order; feeling nervous, uncontrollable worrying, excessive worrying, trouble relaxing, restlessness, irritability, and feeling afraid. The Korean GAD-7 has demonstrated excellent internal consistency across adolescent and adult samples (Cronbach’s α = 0.90–0.93) and strong convergent validity with other validated measures of anxiety and depression [29, 30].
3. *Depressive symptoms and suicidal behaviors* included perceived stress (5-point Likert scale), feelings of sadness or hopelessness, and suicidal ideation, planning, and attempts. The latter four variables were binary and assessed experiences over the past 12 months. These items are standard components of the KYRBS questionnaire and are single-item surveillance indicators; therefore, internal consistency metrics such as Cronbach’s α do not apply. Nonetheless, their reliability is supported by KYRBS test–retest studies [31], and prior research shows that these indicators demonstrate meaningful associations with validated mental-health measures in Korean adolescents [32].
4. *Smartphone overdependence* (SMO) was measured using a 10-item scale developed by Korea’s National Information Society Agency, rated on a 4-point Likert scale (1 = strongly disagree to 4 = strongly agree). Instead of total scores, each item was analyzed individually (SMO 1–10), covering domains such as failed reduction attempts, control difficulty, overuse, preoccupation, physical symptoms, and functional impact. This scale shows strong reliability in Korean adolescent samples (Cronbach’s α = 0.84–0.92) and robust construct validity, with excellent model-fit indices reported in multiple validation studies [33].
5. *Smartphone usage* was measured as self-reported daily average weekend usage time (in hours), reflecting habitual use patterns with minimal influence from the weekday schedule.
6. *Sleep patterns*: KYRBS assesses sleep patterns by asking adolescents to report their usual bedtime and wake time separately for weekdays and weekends. Using these responses, weekday sleep duration was calculated because it reflects regular sleep routines and is less influenced by compensatory oversleep. Weekday sleep onset time was also included.
7. *Substance use* included the lifetime use of alcohol (excluding ceremonial use), tobacco (any product), and narcotics (non-prescribed or illicit drugs), coded as binary variables.

### Statistical analysis

#### Descriptive statistics

Descriptive statistics were calculated to compare variables between the pandemic and post-pandemic samples. Independent t-tests were used for continuous variables and chi-squared tests were used for categorical variables. Given the large sample size, effect sizes (Cohen’s d) were reported along with p-values with thresholds of 0.2, 0.5, and 0.8 indicating small, medium, and large effects, respectively. All analyses were conducted in R (version 4.4.2). Key packages included qgraph (v1.9.8), igraph (version 2.1.2), NetworkComparisonTest (version 2.2.2), bootnet (version 1.6), dplyr (version 1.1.4), ggplot2 (version 3.5.1), glasso (version 1.11) and psych (version 2.4.12).

#### Network estimation

Network structures were estimated using graphical LASSO regularization with extended Bayesian information criterion (EBIC) model selection via the *qgraph* package. We followed standard guidelines for psychological network analysis [34]. The network model is based on Gaussian Graphical Models, which estimate conditional associations through sparse precision matrices [35]. The networks were visualized using the Fruchterman–Reingold algorithm with node positions fixed across timepoints for comparability. Mixed graphical models were used to accommodate the variables of different types, with sex modeled as a binary categorical variable. Although sex appears as a node in the undirected network, it is treated analytically as a demographic attribute rather than a psychological symptom, consistent with its conceptual role described in the Measures section. In this framework, we included sex as a demographic node to quantify population-level differences in the expression and clustering of mental-health variables across groups. Separate sex-stratified networks were additionally estimated to evaluate within-sex symptom configurations, addressing a distinct analytic question from the inclusion of sex in the overall model.

The EBICglasso procedure was used to regularize the precision matrix and select sparse, interpretable structures [36, 37]. The EBIC tuning parameter (γ) was set to 0.5, the default and commonly recommended value in psychological network analysis. To assess robustness, we re-estimated all networks using γ = 0.25 and obtained virtually identical edge weights and centrality metrics, confirming that the results were stable across tuning parameters.

#### Centrality analysis

Centrality indices were estimated to evaluate the relative importance of each node in the network. Following standard definitions in psychological network analysis [34], strength represents the sum of the absolute edge weights connected to a node, indicating its overall level of direct associations within the network. Closeness is defined as the inverse of the average shortest path length to all other nodes, reflecting how efficiently a node can reach the rest of the network. Betweenness captures how often a node lies on the shortest paths between other nodes, indexing its potential role as an intermediary or connector within the system. Case-dropping bootstrap procedures were used to assess the stability and accuracy of centrality estimates.

#### Network comparison

To assess changes in the network structure over time, a network comparison test (NCT) was conducted using the NetworkComparisonTest package. The NCT evaluates the differences in the overall network structure, global strength, and individual edge weights between the pandemic and post-pandemic networks. Statistical significance was assessed using 100 permutations with α = 0.05. False discovery rate (FDR) correction was applied for edge-level comparisons [38].

#### Sex-stratified network analysis

Separate networks were estimated for male and female adolescents to examine sex-based differences in symptom centrality and interconnectivity across the periods.

## Results

### Descriptive statistics

Table 1 presents the comparisons between the pandemic and post-pandemic samples. Although many variables differed significantly due to the large sample sizes, most effect sizes were small (Cohen’s d < 0.1). Notably, suicidal ideation increased from 10.3% to 13.0%, and SMO increased across most items. Seven of ten SMO items had effect sizes > 0.1, with the largest changes seen in “Irritability” (GAD item 6) and “Overuse” (SMO item 3). Weekend smartphone usage increased as well. While the lifetime uses of alcohol and tobacco declined slightly, the rate of narcotics use increased by more than twofold. The detailed descriptive statistics are shown in Table 1.

To contextualize the sex-stratified network findings, Supplementary Table S1 summarizes key descriptive differences between males and females across both periods. Females generally reported higher internalizing symptoms and smartphone overdependence, whereas males showed higher rates of risk-taking behaviors such as alcohol and tobacco use. These quantitative patterns provide a foundation for interpreting the sex-specific centrality differences described later.

### Network structure

**Fig. 1:**
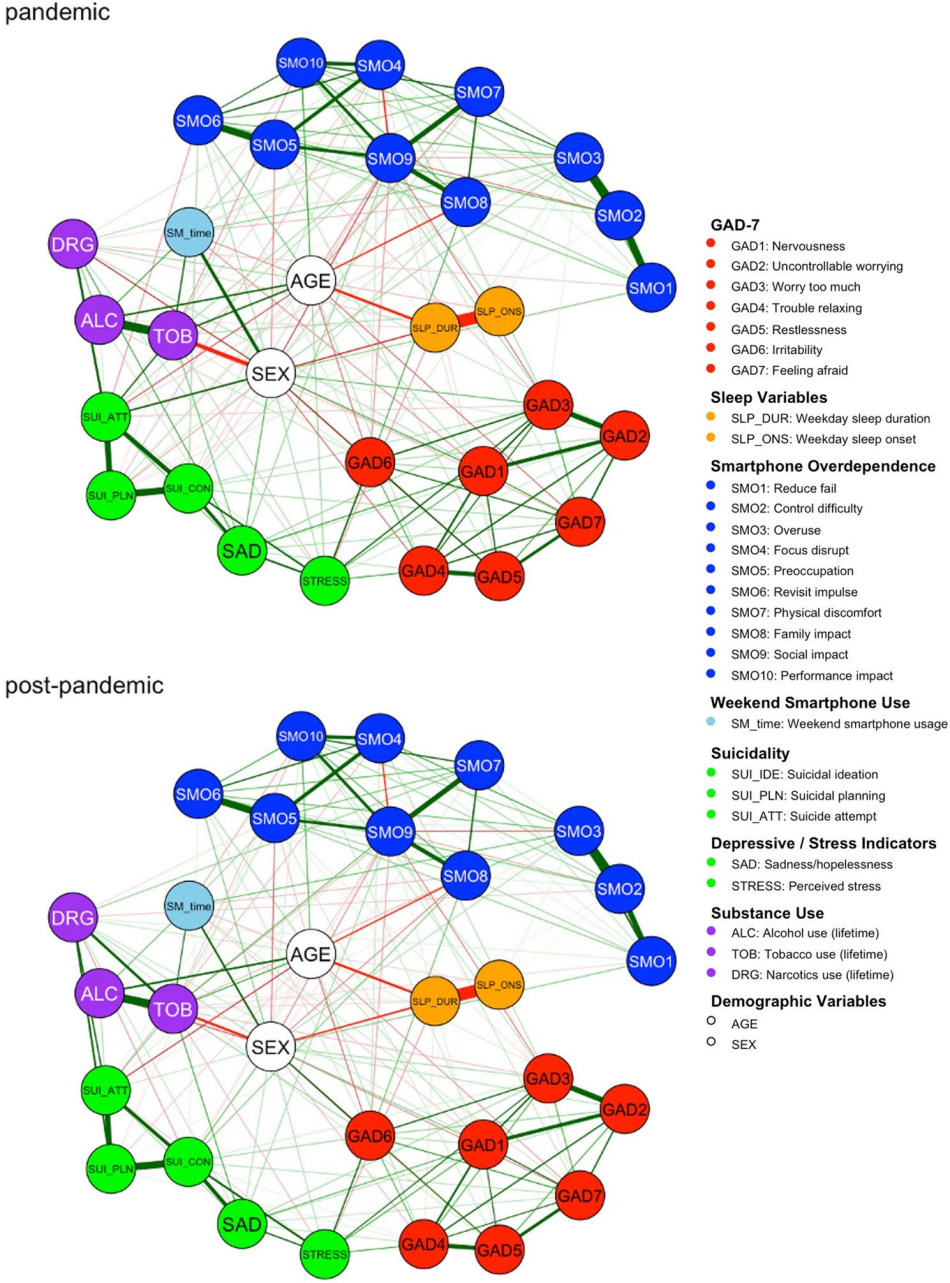
LASSO network structures of the pandemic and post-pandemic period shown with the Fruchterman–Reingold method. Positive and negative correlations are represented each with green and red edges, where thicker edges represent a stronger correlation. EX: female; SMO: smartphone overdependence; SM_time: weekend smartphone usage; SLP_DUR: sleep duration; SLP_ONS: sleep onset; ALC: lifetime experience of alcohol; TOB: lifetime experience of tobacco; DRG: lifetime experience of narcotics; SUI_CON: suicidal ideation; SUI_PLN: suicidal plan; SUI_ATT: suicidal attempt; STRESS: perceived stress; SAD: feeling of sadness or hopelessness; GAD: Generalized Anxiety Disorder 7

The LASSO-regularized networks showed fewer edges in the post-pandemic network (*N* = 188) than in the pandemic network (*N* = 195), indicating reduced symptom connectivity. Figure 1 shows the overall structure for each period using the Fruchterman–Reingold layout. Distinct modular patterns emerged with the GAD-7 and SMO items forming separate clusters. Depression- and suicide-related variables, substance use, and smartphone usage time tended to occupy overlapping regions, indicating interrelationships across the domains. Networks re-estimated using a lower EBIC tuning parameter (γ = 0.25) produced identical edge-weight and centrality patterns, confirming that the results were robust to the choice of γ.

### Network comparison

The NCT revealed a significant difference in the overall network structure between the two timepoints (*p* = 0.010), although global strength was not significantly different (*p* = 0.248). This indicates that the configuration of the connections changed, even when the overall connectivity level remained stable. The complete results of the permutation-based tests are shown in Supplementary Figure S3.

### Edge-level differences between networks

**Fig. 2:**
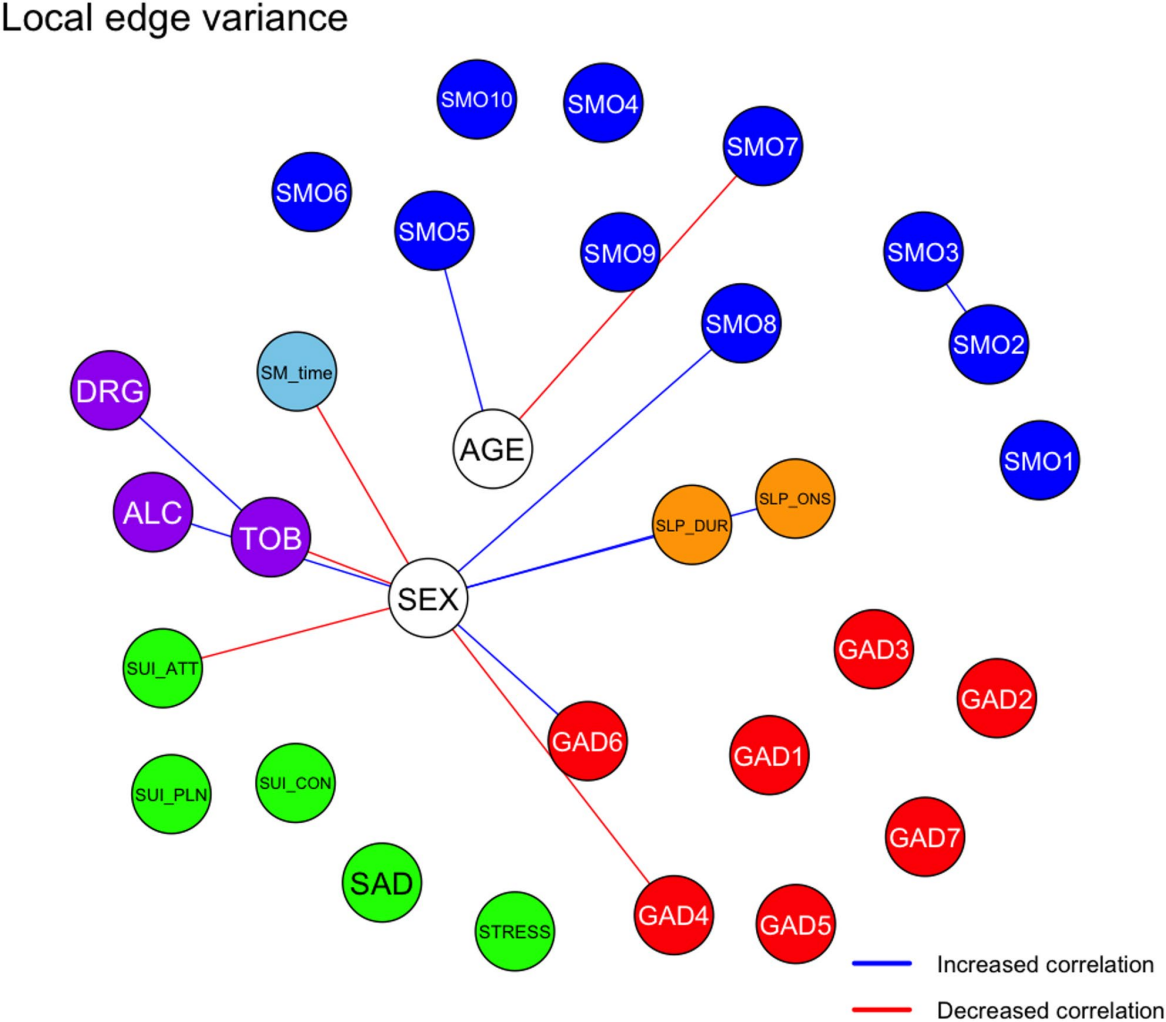
Thirteen edges with significant local difference in the network comparison test are shown, with each increased and decreased correlations shown as blue and red edges. P-values were adjusted with the Benjamini–Hochberg (false discovery rate) method. SEX: female; SMO: smartphone overdependence; SM_time: weekend smartphone usage; SLP_DUR: sleep duration; SLP_ONS: sleep onset; ALC: lifetime experience of alcohol; TOB: lifetime experience of tobacco; DRG: lifetime experience of narcotics; SUI_CON: suicidal ideation; SUI_PLN: suicidal plan; SUI_ATT: suicidal attempt; STRESS: perceived stress; SAD: feeling of sadness or hopelessness; GAD: Generalized Anxiety Disorder 7

To examine the specific structural changes, the edge weight differences between the networks were tested. Fifty-five edges differed significantly at the uncorrected level, with 13 edges remaining significant after FDR correction (Supplementary Tables S3 and S4). The results are shown in Fig. 2.

The most prominent decrease was observed between female sex and suicide attempt (Δr = – 0.111), while the edge between tobacco and narcotics use showed the strongest increase (Δr = + 0.209), suggesting an emergent co-patterning of high-risk behaviors. Female sex was the node with the highest number of altered connections (*N* = 9), including strengthened links with irritability (GAD item 6), sleep variables, family impact of smartphone use, and alcohol use (Supplementary Table 3). These edge-level differences highlighted the specific symptom coactivation patterns that changed between the pandemic and post-pandemic periods.

### Node centrality

**Fig. 3:**
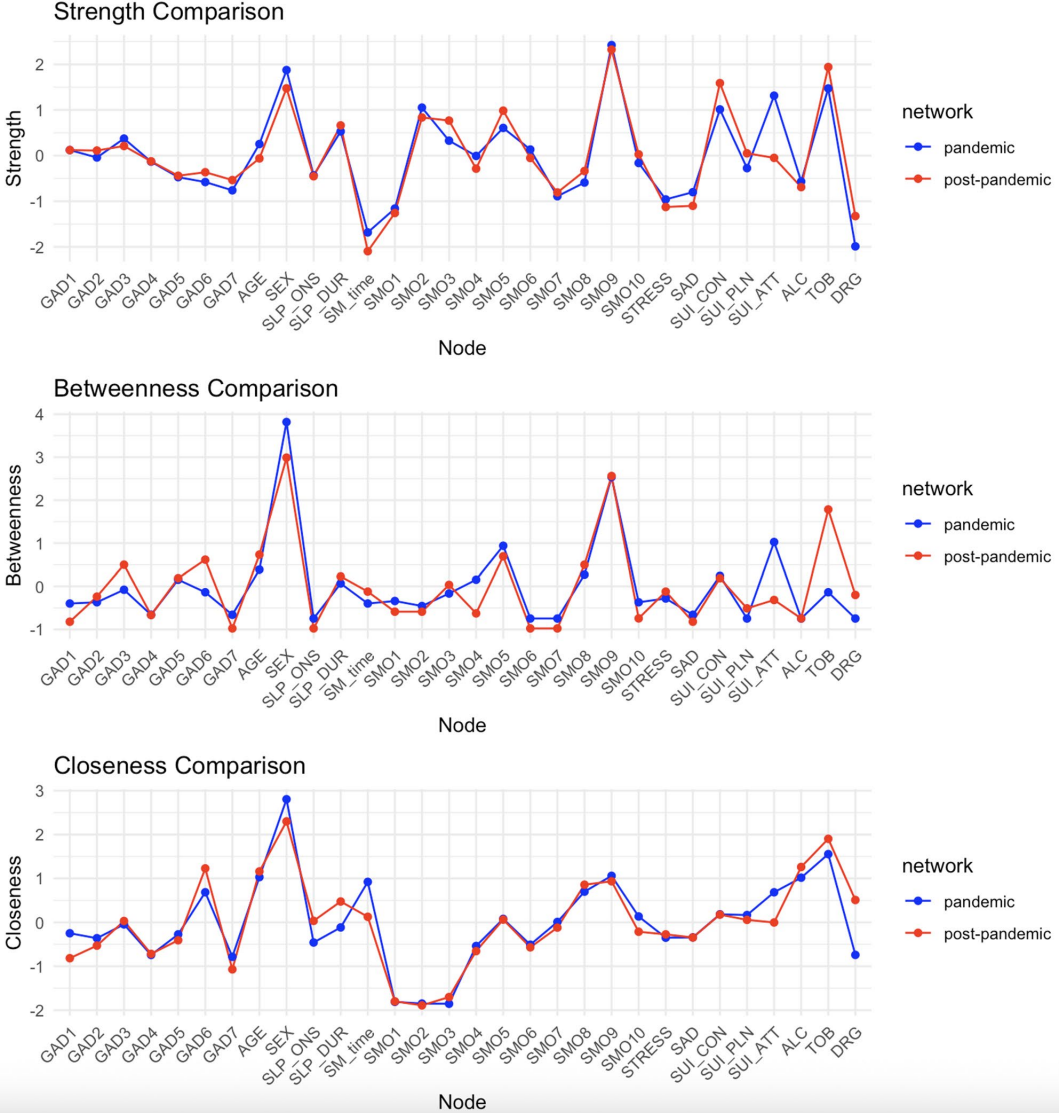
Centrality plots of strength, betweenness, and closeness in the pandemic and post-pandemic periods. SEX, female; SMO, smartphone overdependence; SM_time: weekend smartphone usage; SLP_DUR: sleep duration; SLP_ONS: sleep onset; ALC: lifetime experience of alcohol; TOB: lifetime experience of tobacco; DRG: lifetime experience of narcotics; SUI_CON: suicidal ideation; SUI_PLN: suicidal plan; SUI_ATT: suicidal attempt; STRESS: perceived stress; SAD: feeling of sadness or hopelessness; GAD: Generalized Anxiety Disorder 7

Suicidal ideation and planning exhibited increased strength centrality in the post-pandemic network, especially among females, whereas suicide attempts declined in all centrality metrics. “Irritability” (GAD item 6) showed increased betweenness and closeness centrality, indicating that it occupied a more interconnected position in the post-pandemic network. “Social impact” (SMO item 9) remained the most central node in both periods. Narcotic use and sleep variables also showed increased centrality. Centrality estimates were highly stable (CS coefficient = 0.75); bootstrapped reliability plots are shown in Supplementary Fig. 2a, 2b and Fig. 3.

### Sex-stratified networks

**Fig. 4:**
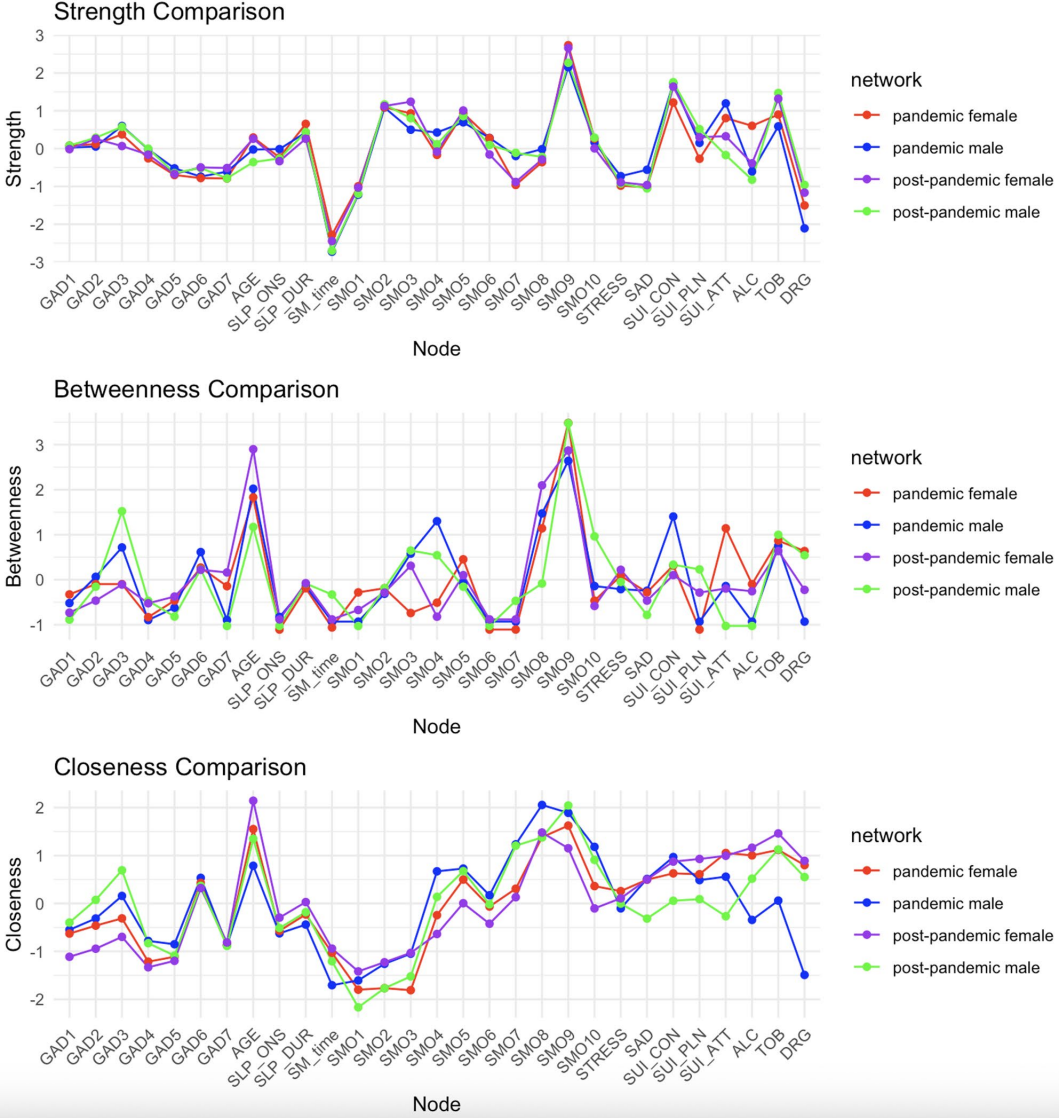
Centrality plots of strength, betweenness, and closeness in males and females in 2020 and 2023. SEX, female; SMO: smartphone overdependence; SM_time: weekend smartphone usage; SLP_DUR: sleep duration; SLP_ONS: sleep onset; ALC: lifetime experience of alcohol; TOB: lifetime experience of tobacco; DRG: lifetime experience of narcotics; SUI_CON: suicidal ideation; SUI_PLN: suicidal plan; SUI_ATT: suicidal attempt; STRESS: perceived stress; SAD: feeling of sadness or hopelessness; GAD: Generalized Anxiety Disorder 7

Sex-stratified networks revealed differences in within-sex centrality patterns. Among females, suicide ideation and planning showed higher centrality, whereas suicide attempts decreased. “Irritability” increased in centrality within female network. Among males, worrying too much (GAD item 3) and trouble relaxing (GAD item 4) showed higher centrality relative to other nodes in the male network. SMO-related items, particularly those reflecting family conflict, showed higher centrality among females, whereas males showed relatively greater centrality in physical/social impact items. Sleep onset became more central among females, whereas sleep duration showed similar patterns across the sexes (Fig. 4).

## Discussion

This study investigated the structural changes in adolescent mental health symptom networks from the pandemic to the post-pandemic period using nationally representative data and a network analysis approach. By focusing on how specific symptoms gained or lost structural prominence, rather than merely tracking prevalence shifts, deeper patterns of psychopathological organization were captured. Although global strength did not differ between periods—indicating comparable overall levels of symptom connectivity—the specific configuration of connections changed substantially. This pattern reflects a reorganization, rather than an intensification, of the mental health network, highlighting shifts in the pathways through which symptoms cluster and reinforce one another. As centrality indices describe the relative interconnectedness of symptoms rather than causal influence, these patterns signal changes in how symptoms co-occurred within adolescents’ post-pandemic experiences. These insights provide a foundation for identifying the symptoms that become most central during periods of rapid social change and for designing more precise, developmentally attuned, and sex-sensitive interventions in the post-pandemic landscape.

Our findings show that, while the prevalence of all suicide-related outcomes increased after the pandemic, only suicidal ideation and planning became more structurally central, especially in female adolescents, whereas suicide attempts declined in centrality. This divergence suggests that although overt suicidal behaviors increased, more internalized forms of suicidality became more tightly embedded within the surrounding symptom structure. These results are consistent with meta-analytic evidence showing a marked increase in suicidal ideation during the pandemic, particularly among adolescent girls [39] and with recent network studies indicating that suicidal ideation has become increasingly embedded within the broader patterns of depression and anxiety [40, 41]. Such changes may reflect altered help-seeking behaviors, intensified emotional distress, and the sustained impact of disruption to social connectedness during the pandemic [42, 43]. Clinically, this structural shift underscores the importance of detecting indirect or internalized suicide risk signals. The rising centrality of suicidal ideation and planning calls for early sex-sensitive screening and preventive strategies that target the pre-attempt phase of suicidality.

Additionally, the impact of SMO on families occupied a more central position in the post-pandemic network, particularly among girls. This pattern reflects stronger associations among device-related family conflict, irritability, and other mental-health variables, rather than implying directional influence. Previous studies have shown that family support is a strong protective factor, and girls are particularly vulnerable to disruptions in their primary support systems [44]. Empirical findings from Korea and other contexts also indicate that poor parent–child relationships, negative parenting practices, and parents’ own problematic smartphone use are associated with higher levels of adolescent overdependence [45]. Together with evidence associating high digital engagement to persistent emotional difficulties during the pandemic [11, 12], our findings underscore the need for interventions that go beyond individual behavioral modifications to address the family context. Strengthening parent–child communication and promoting consistent, supportive parenting practices may be particularly beneficial for female adolescents.

Another key finding was the developmental variation in smartphone use. Several overdependence items (e.g., overuse, family impact, and performance impact) shifted across periods and sexes, suggesting that not all aspects of digital engagement changed uniformly. Previous studies have shown that younger adolescents are more prone to compulsive or solitary use, whereas older adolescents engage in longer sessions and rely more on social and peer-related functions [46–48]. These developmental trajectories imply that certain aspects of smartphone use may become salient at different stages of adolescence. Future research should clarify how these age-specific patterns interact with mental health outcomes to inform developmentally sensitive preventive strategies.

We also observed sex-specific reorganization of anxiety networks during the pandemic transition. Within the female network, irritability (GAD item 6) occupied a relatively more central position, reflecting its greater interconnectedness with other anxiety-related symptoms. Within the male network, worrying too much (GAD item 3) and trouble relaxing (GAD item 4) showed higher relative interconnectedness, indicating that cognitive aspects of worry were more embedded in the male symptom structure. While large-scale studies have consistently shown a higher overall prevalence and severity of anxiety in girls [49], our findings extend this evidence by demonstrating that the configuration of anxiety symptoms shifted differently for boys and girls after the pandemic. These sex-specific reorganizations highlight the importance of moving beyond overall symptom counts to examine structural differences. They also underscore the need for sex-sensitive approaches that target irritability and emotional regulation in girls, and worry management and relaxation strategies in boys, particularly in the evolving post-pandemic environment.

Sleep-related problems became more common after the pandemic, with stronger associations among girls. Both delayed sleep onset and shorter duration showed increased centrality, indicating that sleep disturbances were more interconnected with other symptoms among female adolescents. These findings align with epidemiological evidence showing that insomnia symptoms emerge more frequently in girls during early to mid-adolescence, often coinciding with pubertal timing and hormonal changes [50, 51]. Importantly, meta-analytic evidence links adolescent sleep disturbances with suicidal thoughts and behaviors [13, 52], underscoring the clinical relevance of sleep as a transdiagnostic risk factor. Together, these results indicate that post-pandemic shifts in sleep regulation may disproportionately burden female adolescents, highlighting the importance of monitoring and addressing sleep problems as a core component of post-pandemic mental health strategies.

Substance use patterns also changed after the pandemic. While alcohol and tobacco use declined slightly, narcotics use exhibited more than twofold rise, and its connections with other behaviors strengthened. In particular, the tobacco–narcotics association increased, suggesting that risky behaviors are becoming more clustered rather than isolated. This emerging co-patterning is consistent with prior research showing that early use of one substance often lowers barriers to experimenting with others and accelerates the progression to polysubstance involvement [53]. From clinical and public health perspectives, these findings underscore the importance of surpassing single-substance prevention strategies. Screening and interventions should monitor behavior combinations, as shifts in drugs co-use networks may serve as early warning signs of growing vulnerability in adolescents, particularly in the evolving post-pandemic settings.

Beyond individual symptom changes, broader post-pandemic contextual shifts may help explain the observed network reorganization. With the resumption of offline classes, adolescents faced a rapid return to academic pressure, competitive school environments, and increased social comparison—factors known to heighten internalizing symptoms. Family routines also re-stabilized, accompanied by strengthened parental monitoring, which may have contributed to the enhanced links among irritability, sleep disruption, and smartphone-related family conflict. In parallel, reduced social isolation but heightened performance expectations may have facilitated the shift from behavioral manifestations (e.g., suicide attempts) toward more internalized forms of suicidality becoming structurally central. These transitions provide a broader contextual framework for the symptom clustering patterns observed in the post-pandemic networks.

These patterns are further shaped by the sociocultural context of South Korean adolescents. Persistent academic competitiveness, long study hours, and widespread private tutoring may intensify vulnerability to sleep restriction and irritability, which aligns with the strengthened sleep–irritability–conflict pathways observed among girls. In addition, collectivist family norms and strong parent–child interdependence can magnify emotional responses to family tension, while strict parental control over smartphone use may exacerbate device-related conflict. Gendered expectations that discourage emotional expression among boys may channel distress toward cognitive forms of anxiety, consistent with the increased centrality of worry-related symptoms. Together, these cultural dynamics complement the social-contextual explanation for the sex-differentiated networks identified in this study.

Taken together, our findings demonstrate that the pandemic and its aftermath have reorganized the structure of adolescent mental health symptoms in meaningful ways. The shifts observed in suicidality, anxiety profiles, smartphone-related family conflict, sleep integration, and clustering of substance use suggest that rapid and large-scale interferences can alter not only the prevalence but also the architecture of psychopathology in youth. This perspective extends beyond COVID-19 pandemic itself. Future pandemics or comparable global crises are likely to generate similar reconfigurations, particularly in vulnerable populations such as adolescents. Recognizing which symptoms become central during such transitions can provide early, developmentally tailored, and sex-sensitive interventions.

Despite its strengths, this study had several limitations. First, the data were cross-sectional, which constrained causal inferences regarding the observed changes across time points. Because KYRBS is a repeated cross-sectional survey with different participants sampled each year, within-person temporal dynamics or causal directions cannot be assessed; future studies using true longitudinal or panel designs are needed to trace how adolescent mental health networks change over time. Second, the use of self-reported measures raises the possibility of reporting bias, particularly for sensitive topics such as suicidality and substance use. Third, although network analysis provides unique insights into symptom structures, it does not establish directionality or causality, and the centrality indices can be influenced by network size and density; interpretations of centrality in this study therefore refer solely to patterns of interconnectedness within the observed networks. These methodological considerations should be acknowledged when interpreting our study results.

In conclusion, this study provides novel evidence that adolescent mental health networks underwent structural reorganization in the wake of the pandemic, with distinct patterns according to sex and symptom domains. These findings highlight the value of a network perspective for detecting early warning signs and guiding preventive efforts not only in the context of COVID-19 pandemic but also in preparing for future crises that may similarly disrupt the developmental trajectories of young people.

**Table 1:** Sample characteristics and comparisons between pandemic and post-pandemic periods

	Pandemic (*N* = 46387)	Post-pandemic (*N* = 48936)	Mean diff % Diff	t $\:{\boldsymbol{\chi\:}}^{2}$	*p*-value	Effect size (Cohen’s d or φ)
Female sex, N(%)	22,283 (48.0%)	24,376 (49.8%)	+ 1.8%	29.95	< 0.001	0.02
Age (years)	15.1 (1.8)	15.1 (1.7)	-0.04	3.62	< 0.001	0.023
Onset of sleep (h)	24.8 (1.5)	24.8 (1.4)	-0.05	5.38	< 0.001	0.035
Sleep duration (h)	6.3 (1.5)	6.3 (1.4)	0.00	0.08	0.935	< 0.01
Perceived stress: 0 ~ 4	3.2 (0.9)	3.3 (0.9)	0.10	16.23	< 0.001	0.105
Feeling of sadness or hopelessness, N (%)	11,222 (24.2%)	12,529 (25.6%)	+ 1.4%	25.26	< 0.001	0.03
Suicidal ideation, N (%)	4775 (10.3%)	6354 (13.0%)	+ 2.7%	166.9	< 0.001	0.04
Suicidal plan, N (%)	1494 (3.2%)	2383 (4.9%)	+ 1.6%	165.52	< 0.001	0.04
Suicidal attempt, N (%)	826 (1.8%)	1372 (2.8%)	+ 1.0%	110.18	< 0.001	0.03
Lifetime experience for alcohol, N (%)	15,118 (32.6%)	15,745 (32.2%)	-0.4%	1.87	0.172	< 0.01
Lifetime experience for tobacco, N (%)	4735 (10.2%)	4652 (9.5%)	-0.7%	13.11	< 0.001	0.01
Lifetime experience for narcotics, N (%)	291 (0.6%)	661 (1.4%)	+ 0.7%	125.31	< 0.001	0.04
GAD-7 scores (0–21)	3.827 (4.271)	4.151 (4.500)	+ 0.324	11.395	< 0.001	0.074
Weekend smartphone usage (min)	381.7 (243.8)	390.4 (228.4)	+ 8.71	5.68	< 0.001	0.037
Smartphone overdependence (10–40)	18.385 (6.150)	19.217 (6.056)	+ 0.833	21.05	< 0.001	0.14

GAD-7: 7-item Generalized Anxiety Disorder scale

## Supplementary Information

Below is the link to the electronic supplementary material.

Supplementary Material 1.

## Data Availability

All KYRBS data are available to the public and can be downloaded from the KYRBS official website (https://www.kdca.go.kr/yhs/).
