# Supplementary material for: Structural changes in adolescent mental health networks from the pandemic to the post-pandemic period: a network comparison study
Source: Child Adolesc Psychiatry Ment Health. 2026 Jan 31;20:30. doi: 10.1186/s13034-025-01021-0 (PMC12947468; doi:10.1186/s13034-025-01021-0)
Supplement: Supplementary file 1 — Supplementary Material 1. [file 13034_2025_1021_MOESM1_ESM.docx]

**Figure S1.** Flowchart of participant inclusion and exclusion for the 2020 and 2023 KYRBS datasets.


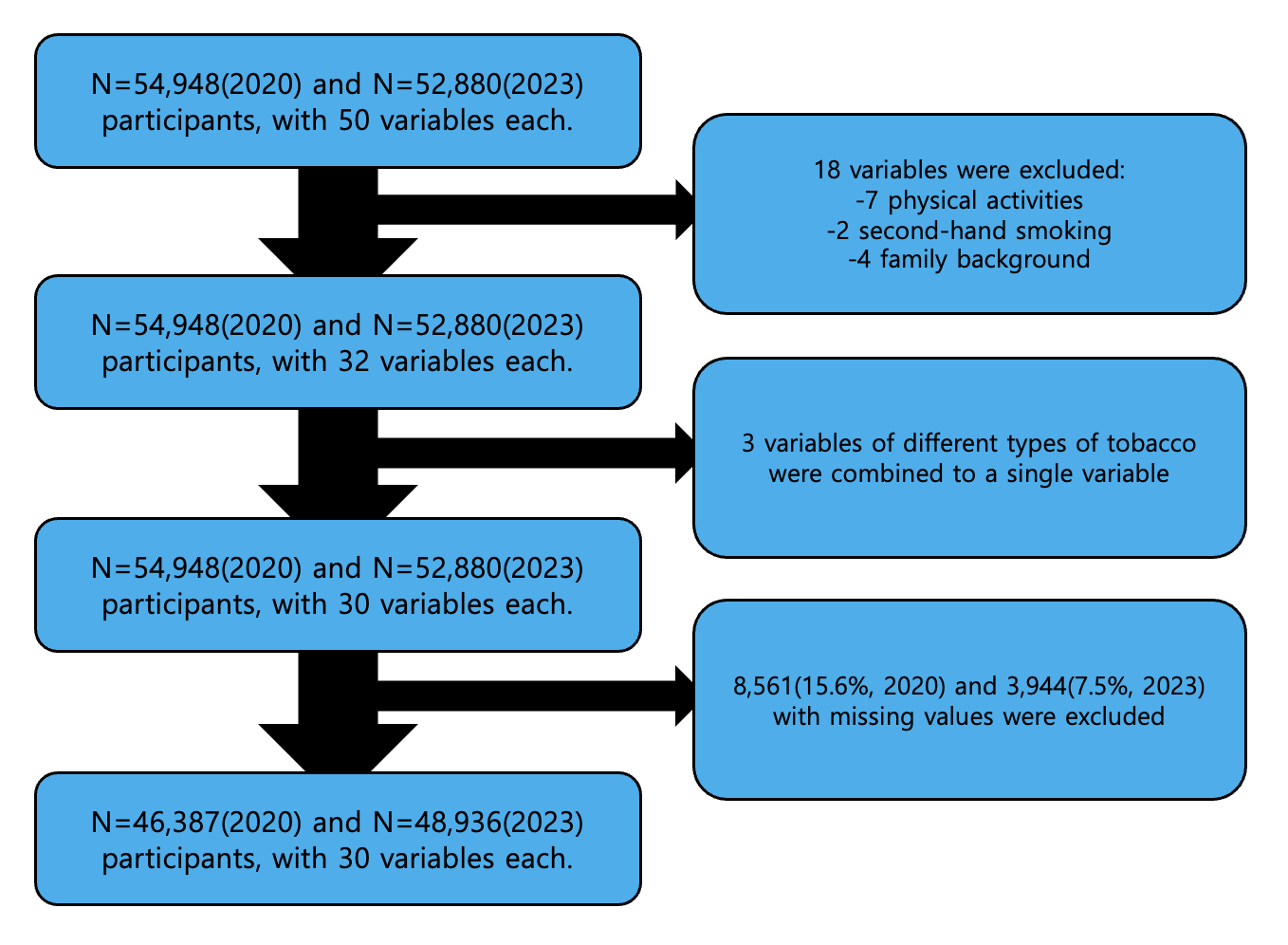


Figure S2a. Stability of the pandemic network structure.


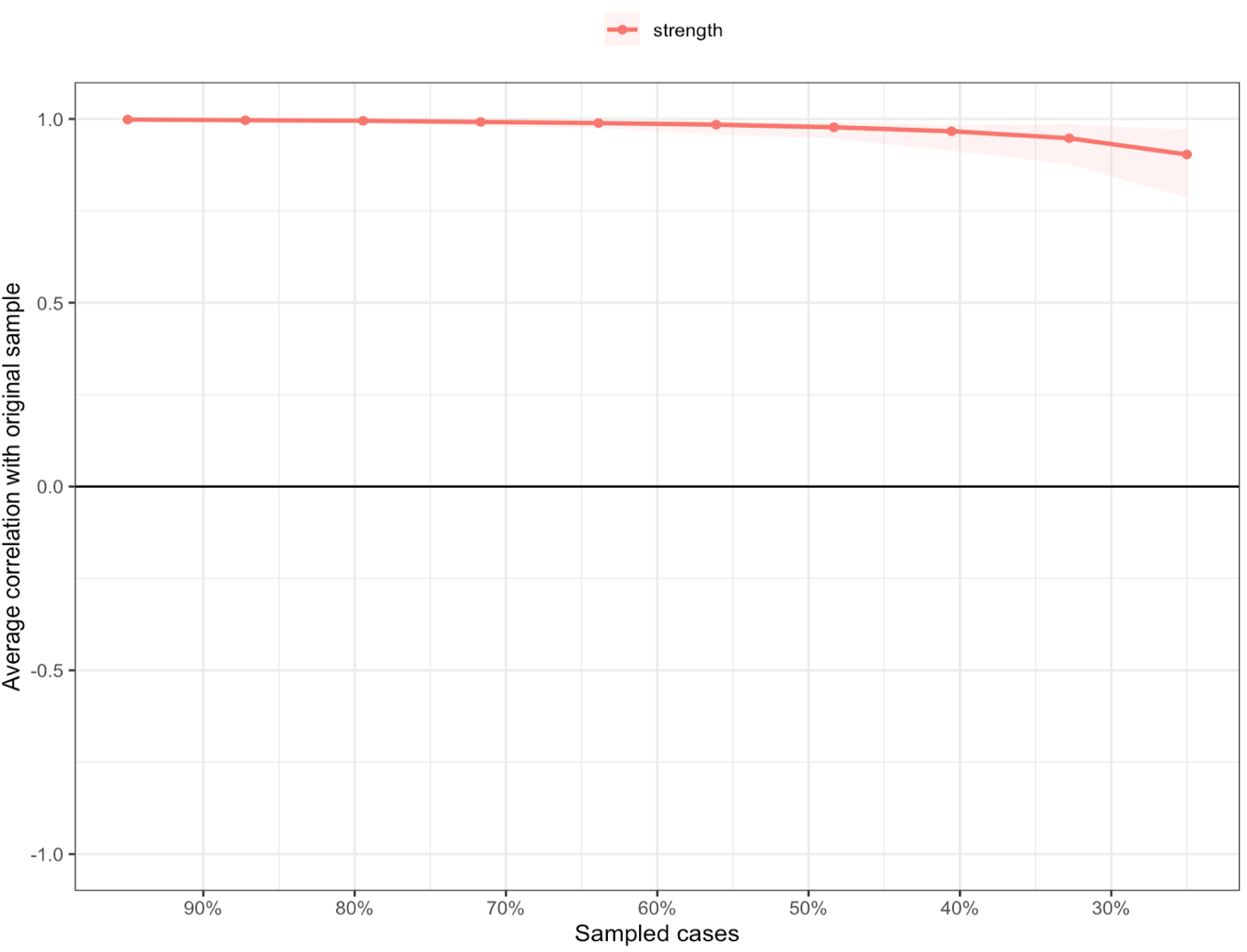


Figure S2b. Stability of post-pandemic network structure.


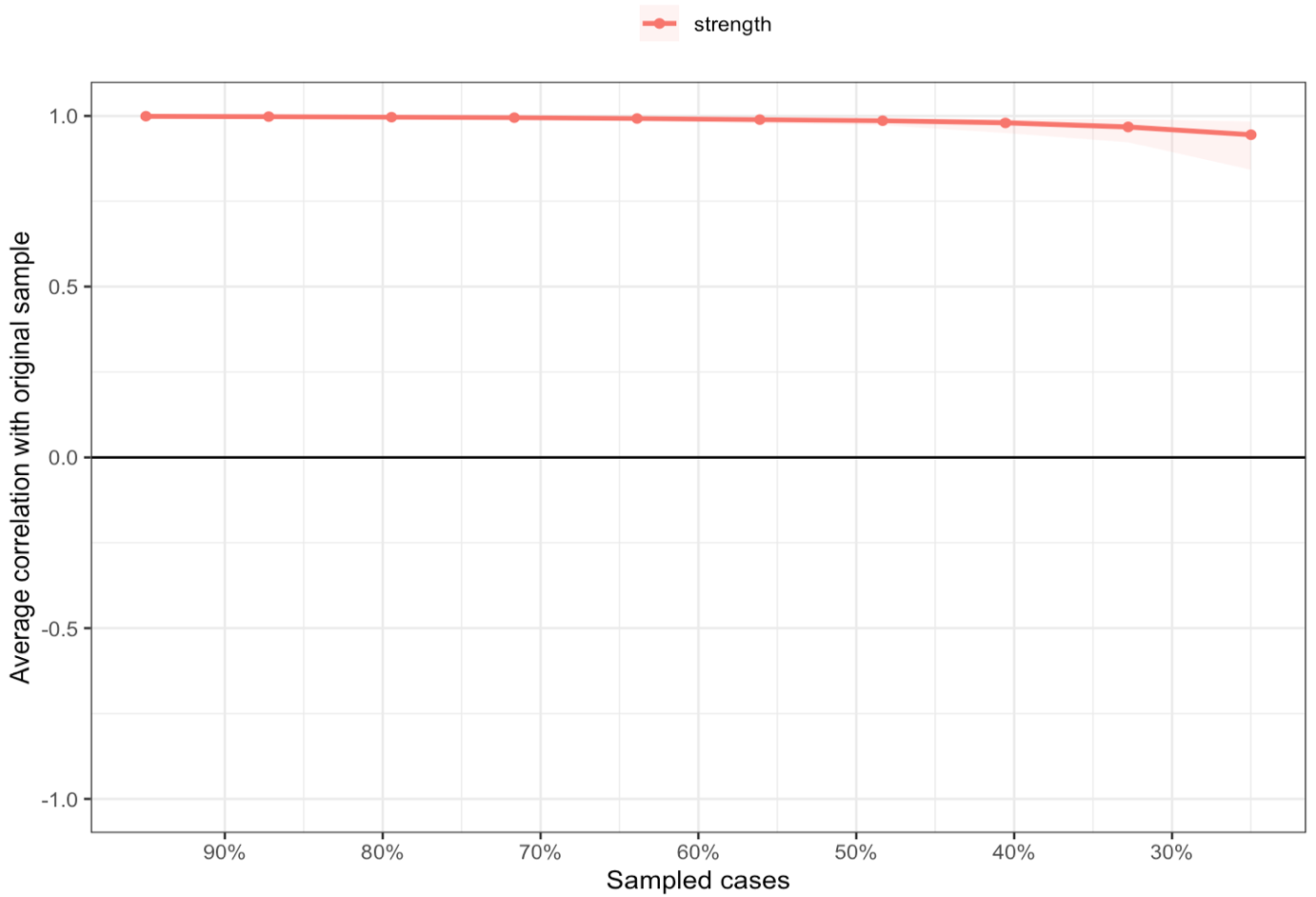


Figure S3. Network comparison test of pandemic vs post-pandemic. NCT function of the NetworkComparisonTest package was used. Each present NCT results of network structure, global strength, and global edge invariance.


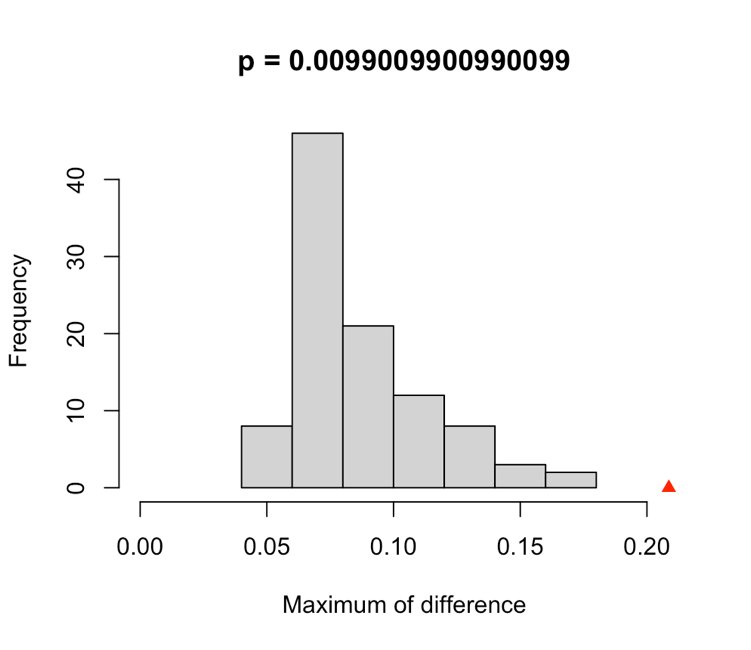

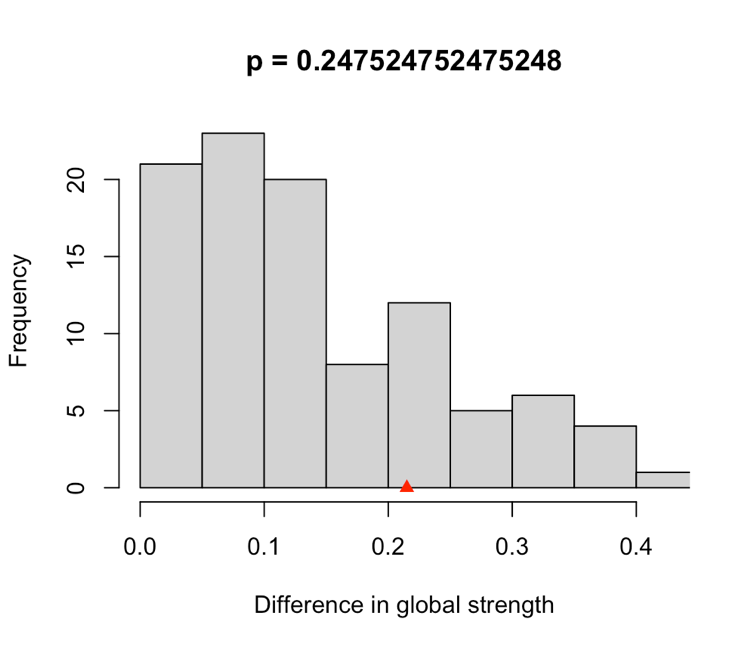


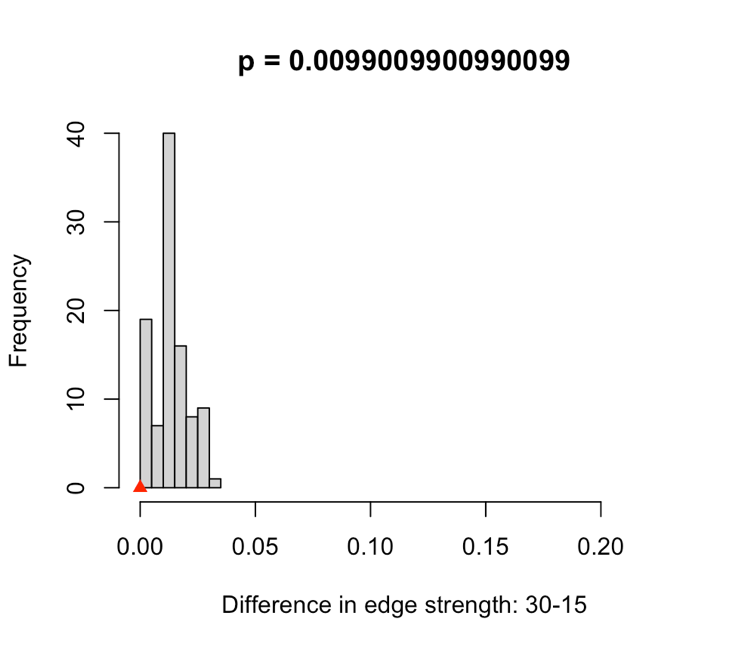


**Table S1.** Descriptive statistics of key variables stratified by gender across pandemic (2020), post-pandemic (2023), and overall samples.

|  | **2020 Pandemic** | | **2023 Post-pandemic** | | **Overall** | |
| --- | --- | --- | --- | --- | --- | --- |
|  | **Male (N=24104)** | **Female (N=22283)** | **Male (N=24560)** | **Female (N=24376)** | **Male (N=48664)** | **Female (N=46659)** |
| **Age (years)** | 15.1 (1.8) | 15.1 (1.8) | 15.1 (1.7) | 15.1 (1.7) | 15.1 (1.8) | 15.1 (1.7) |
| **Onset of sleep (h)** | 24.6 (1.4) | 25.0 (1.5) | 24.6 (1.4) | 24.9 (1.4) | 24.6 (1.4) | 25.0 (1.4) |
| **Sleep duration (h)** | 6.5 (1.5) | 6.0 (1.5) | 6.5 (1.4) | 6.0 (1.4) | 6.5 (1.4) | 6.0 (1.5) |
| **Perceived stress: 0~4** | 3.0 (0.9) | 3.3 (0.9) | 3.1 (0.9) | 3.4 (0.9) | 3.0 (0.9) | 3.4 (0.9) |
| **Feeling of sadness or hopelessness, N (%)** | 4620 (19.2%) | 6602 (29.6%) | 5148 (21.0%) | 7381 (30.3%) | 9768 (20.1%) | 13983 (30.0%) |
| **Suicidal ideation, N (%)** | 1817 (7.5%) | 2958 (13.3%) | 2261 (9.2%) | 4093 (16.8%) | 4078 (8.4%) | 7051 (15.1%) |
| **Suicidal plan, N (%)** | 585 (2.4%) | 909 (4.1%) | 880 (3.6%) | 1503 (6.2%) | 1465 (3.0%) | 2412 (5.2%) |
| **Suicidal attempt, N (%)** | 272 (1.1%) | 554 (2.5%) | 494 (2.0%) | 878 (3.6%) | 766 (1.6%) | 1432 (3.1%) |
| **Lifetime experience for alcohol, N (%)** | 8783 (36.4%) | 6335 (28.4%) | 9109 (37.1%) | 6636 (27.2%) | 17892 (36.8%) | 12971 (27.8%) |
| **Lifetime experience for tobacco, N (%)** | 3345 (13.9%) | 1390 (6.2%) | 3041 (12.4%) | 1611 (6.6%) | 6386 (13.1%) | 3001 (6.4%) |
| **Lifetime experience for narcotics, N (%)** | 161 (0.7%) | 130 (0.6%) | 293 (1.2%) | 368 (1.5%) | 454 (0.9%) | 498 (1.1%) |
| **GAD-7 scores (0–21)** | 3.085 (3.891) | 4.629 (4.512) | 3.331 (4.096) | 4.977 (4.732) | 3.209 (3.998) | 4.811 (4.632) |
| **Weekend smartphone usage (min)** | 334.4 (230.7) | 432.8 (247.2) | 358.2 (226.2) | 422.8 (226.0) | 346.4 (228.7) | 427.6 (236.4) |
| **Smartphone overdependence (10–40)** | 17.514 (6.137) | 19.326 (6.025) | 18.488 (6.108) | 19.953 (5.913) | 18.006 (6.142) | 19.653 (5.975) |

Values are presented as mean (standard deviation) or number (%). Sleep onset time is coded on a 0–24 hour scale.

Table S2. Item-level comparisons of GAD-7 and SMO of pandemic, post-pandemic.

|  | **Pandemic (N=46387)** | **Post-pandemic (N=48936)** | **Mean diff**  **% Diff** | **t**  $\boldsymbol{\chi}^{\boldsymbol{2}}$ | **p-value** | **Effect size**  **(Cohen’s d or φ)** |
| --- | --- | --- | --- | --- | --- | --- |
| **GAD-7 scores: 0~3** |  |  |  |  |  |  |
| **GAD 1**  **: Nervousness** | 0.549 (0.752) | 0.600 (0.783) | +0.050 | 10.15 | <0.001 | 0.066 |
| **GAD 2**  **: Uncontrollable worrying** | 0.584 (0.808) | 0.632 (0.842) | +0.048 | 8.92 | <0.001 | 0.058 |
| **GAD 3**  **: Worry too much** | 0.963 (0.944) | 0.994 (0.954) | +0.031 | 5.09 | <0.001 | 0.033 |
| **GAD 4**  **: Trouble relaxing** | 0.445 (0.751) | 0.493 (0.787) | +0.048 | 9.58 | <0.001 | 0.062 |
| **GAD 5**  **: Restlessness** | 0.264 (0.602) | 0.300 (0.646) | +0.037 | 9.03 | <0.001 | 0.058 |
| **GAD 6**  **: Irritability** | 0.678 (0.852) | 0.755 (0.892) | +0.077 | 13.57 | <0.001 | 0.088 |
| **GAD 7**  **: Feeling afraid** | 0.343 (0.697) | 0.377 (0.732) | +0.033 | 7.22 | <0.001 | 0.047 |
| **Smartphone Overdependence: 1~4** |  |  |  |  |  |  |
| **SMO 1**  **: Reduce fail** | 2.116 (0.882) | 2.218 (0.868) | +0.102 | 17.98 | <0.001 | 0.116 |
| **SMO 2**  **: Control difficulty** | 2.140 (0.903) | 2.254 (0.886) | +0.114 | 19.63 | <0.001 | 0.127 |
| **SMO 3**  **: Overuse** | 2.138 (0.903) | 2.261 (0.887) | +0.123 | 21.19 | <0.001 | 0.137 |
| **SMO 4**  **: Focus disrupt** | 2.101 (0.913) | 2.188 (0.901) | +0.087 | 14.76 | <0.001 | 0.096 |
| **SMO 5**  **: Preoccupation** | 1.658 (0.742) | 1.747 (0.763) | +0.089 | 18.34 | <0.001 | 0.119 |
| **SMO 6**  **: Revisit impulse** | 1.784 (0.817) | 1.873 (0.830) | +0.089 | 16.78 | <0.001 | 0.109 |
| **SMO 7**  **: Physical discomfort** | 1.568 (0.743) | 1.582 (0.735) | +0.014 | 2.94 | 0.003 | 0.019 |
| **SMO 8**  **: Family impact** | 1.740 (0.844) | 1.833 (0.876) | +0.094 | 16.84 | <0.001 | 0.109 |
| **SMO 9**  **: Social impact** | 1.370 (0.579) | 1.414 (0.605) | +0.045 | 11.71 | <0.001 | 0.076 |
| **SMO 10**  **: Performance impact** | 1.770 (0.837) | 1.846 (0.855  ) | +0.076 | 13.81 | <0.001 | 0.089 |

Table S3. 55 edges showing a significant difference (p<0.05) in local edge weight of the network comparison test, with no adjustment in p-values.

| **No.** | **Node 1** | **Node 2** | **p-value** | **Change of correlation** |
| --- | --- | --- | --- | --- |
| 1 | Uncontrollable worrying (GAD 2) | Worry too much (GAD 3) | <0.001 | +0.024 |
| 2 | Trouble relaxing (GAD 4) | Female sex (SEX) |  | -0.028 |
| 3 | Irritability (GAD 6) | Female sex (SEX) |  | +0.045 |
| 4 | Female sex (SEX) | Sleep onset (SLP_ONS) |  | +0.045 |
| 5 | Female sex (SEX) | Sleep duration (SLP_DUR) |  | +0.042 |
| 6 | Female sex (SEX) | Weekend smartphone usage (SM_time) |  | -0.084 |
| 7 | Control difficulty (SMO 2) | Overuse (SMO 3) |  | +0.034 |
| 8 | Age | Preoccupation (SMO 5) |  | +0.023 |
| 9 | Age | Physical discomfort (SMO 7) |  | -0.037 |
| 10 | Female sex (SEX) | Lifetime experience for alcohol (ALC) |  | +0.071 |
| 11 | Female sex (SEX) | Lifetime experience for tobacco (TOB) |  | -0.099 |
| 12 | Female sex (SEX) | Lifetime experience for narcotics (DRG) |  | -0.057 |
| 13 | Lifetime experience for tobacco (TOB) | Lifetime experience for narcotics (DRG) |  | +0.209 |
| 14 | Feeling afraid (GAD 7) | Female sex (SEX) | 0.002 | +0.026 |
| 15 | Lifetime experience for alcohol (ALC) | Lifetime experience for tobacco (TOB) |  | -0.037 |
| 16 | Suicidal plan (SUI_PLN) | Lifetime experience for narcotics (DRG) |  | +0.160 |
| 17 | Female sex (SEX) | Suicidal attempt (SUI_ATT) | 0.003 | -0.111 |
| 18 | Female sex (SEX) | Family impact (SMO 8) | 0.004 | +0.049 |
| 19 | Revisit impulse (SMO 6) | Suicidal attempt (SUI_ATT) |  | -0.036 |
| 20 | Weekend smartphone usage (SM_time) | Overuse (SMO 3) | 0.005 | +0.018 |
| 21 | Sleep onset (SLP_ONS) | Lifetime experience for tobacco (TOB) | 0.006 | -0.032 |
| 22 | Nervousness (GAD 1) | Weekend smartphone usage (SM_time) | 0.007 | -0.018 |
| 23 | Female sex (SEX) | Feeling of sadness or hopeless (SAD) |  | -0.038 |
| 24 | Female sex (SEX) | Physical discomfort (SMO 7) | 0.010 | -0.020 |
| 25 | Overuse (SMO 3) | Social impact (SMO 9) |  | +0.035 |
| 26 | Worry too much (GAD 3) | Female sex (SEX) | 0.011 | -0.023 |
| 27 | Age | Perceived stress (STRESS) |  | -0.036 |
| 28 | Nervousness (GAD 1) | Perceived stress (STRESS) | 0.012 | +0.019 |
| 29 | Family impact (SMO 8) | Social impact (SMO 9) | 0.013 | -0.024 |
| 30 | SMO 4 (Focus disrupt) | Suicidal plan (SUI_PLN) |  | -0.024 |
| 31 | Age | Lifetime experience for tobacco (TOB) | 0.014 | -0.037 |
| 32 | Reduce fail (SMO 1) | Lifetime experience for narcotics (DRG) |  | -0.006 |
| 33 | Feeling afraid (GAD 7) | Age | 0.017 | -0.015 |
| 34 | Sleep duration (SLP_DUR) | Suicidal attempt (SUI_ATT) |  | -0.026 |
| 35 | Nervousness (GAD 1) | Feeling afraid (GAD 7) | 0.018 | +0.023 |
| 36 | Female sex (SEX) | Suicidal ideation (SUI_CON) |  | +0.059 |
| 37 | Nervousness (GAD 1) | Female sex (SEX) | 0.021 | -0.021 |
| 38 | Age | Revisit impulse (SMO 6) |  | -0.016 |
| 39 | Overuse (SMO 3) | Family impact (SMO 8) |  | +0.028 |
| 40 | Physical d(SMO 7) | Suicidal plan (SUI_PLN) | 0.022 | -0.018 |
| 41 | Worry too much (GAD 3) | Trouble relaxing (GAD 4) | 0.025 | -0.018 |
| 42 | Perceived stress (STRESS) | Suicidal attempt (SUI_ATT) |  | +0.001 |
| 43 | Physical discomfort (SMO 7) | Lifetime experience for tobacco (TOB) | 0.026 | +0.026 |
| 44 | Worry too much (GAD 3) | Lifetime experience for tobacco (TOB) | 0.027 | +0.026 |
| 45 | Social impact (SMO 9) | Lifetime experience for tobacco (TOB) | 0.031 | +0.037 |
| 46 | Feeling of sadness or hopeless (SAD) | Suicidal ideation (SUI_CON) | 0.032 | +0.043 |
| 47 | Reduce fail (SMO 1) | Control difficulty (SMO 2) | 0.034 | -0.122 |
| 48 | Sleep onset (SLP_ONS) | Control difficulty (SMO 2) | 0.035 | +0.005 |
| 49 | SMO 4 (Focus disrupt) | Lifetime experience for narcotics (DRG) | 0.037 | -0.015 |
| 50 | Female sex (SEX) | Suicidal plan (SUI_PLN) | 0.038 | -0.031 |
| 51 | Reduce fail (SMO 1) | Overuse (SMO 3) | 0.039 | +0.073 |
| 52 | Female sex (SEX) | Revisit impulse (SMO 6) | 0.042 | +0.021 |
| 53 | Age | Lifetime experience for alcohol (ALC) | 0.045 | +0.025 |
| 54 | Irritability (GAD 6) | Lifetime experience for tobacco (TOB) |  | +0.027 |
| 55 | Uncontrollable worrying (GAD 2) | Irritability (GAD 6) | 0.049 | +0.017 |

Table S4. 13 edges showing a significant difference (p<0.05) in local edge weight of the network comparison test, with p-values adjusted with the Benjamini-Hochberg(FDR) method. “Change of correlation” is derived from the difference of absolute values of the correlations.

| **No.** | **Node 1** | **Node 2** | **Change of correlation** |
| --- | --- | --- | --- |
| 1 | GAD 4 (Trouble relaxing) | Female sex (SEX) | -0.028 |
| 2 | GAD 6 (Irritability) | Female sex (SEX) | +0.045 |
| 3 | Female sex (SEX) | Sleep onset (SLP_ONS) | +0.045 |
| 4 | Female sex (SEX) | Sleep duration (SLP_DUR) | +0.042 |
| 5 | Female sex (SEX) | Weekend smartphone usage (SM_time) | -0.084 |
| 6 | SMO 2 (Control difficulty) | SMO 3 (Overuse) | +0.034 |
| 7 | Age | SMO 5 (Preoccupation) | +0.023 |
| 8 | Age | SMO 7 (Physical discomfort) | -0.037 |
| 9 | Female sex (SEX) | SMO 8 (Family impact) | +0.049 |
| 10 | Female sex (SEX) | Suicidal attempt (SUI_ATT) | -0.111 |
| 11 | Female sex (SEX) | Lifetime experience for alcohol (ALC) | +0.071 |
| 12 | Female sex (SEX) | Lifetime experience for tobacco (TOB) | -0.099 |
| 13 | Lifetime experience for tobacco (TOB) | Lifetime experience for narcotics (DRG) | +0.209 |

Table S5. Number of edges with a significant difference (p<0.05) in local edge weight of the network comparison test, that are connected to each nodes. P-values adjusted with the Benjamini-Hochberg (FDR) method.

| **Node** | **Number of attached edges with a significant difference in local edge weight** |
| --- | --- |
| Female sex (SEX) | 9 |
| Age | 2 |
| Lifetime experience for tobacco (TOB) |  |
| Trouble relaxing (GAD 4) | 1 |
| Irritability (GAD 6) |  |
| Sleep onset (SLP_ONS) |  |
| Sleep duration (SLP_DUR) |  |
| Weekend smartphone usage (SM_time) |  |
| Control difficulty (SMO 2) |  |
| Overuse (SMO 3) |  |
| Preoccupation (SMO 5) |  |
| Physical discomfort (SMO 7) |  |
| Family impact (SMO 8) |  |
| Lifetime experience for narcotics (DRG) |  |
| Lifetime experience for alcohol (ALC) |  |
| Suicidal attempt (SUI_ATT) |  |
